# Home and Community Environmental Features, Activity Performance, and Community Participation among Older Adults with Functional Limitations

**DOI:** 10.1155/2012/625758

**Published:** 2011-11-16

**Authors:** Hsiang-Yu Yang, Jon A. Sanford

**Affiliations:** Center for Assistive Technology and Environmental Access (CATEA), Georgia Institute of Technology, Atlanta, GA 30318, USA

## Abstract

This paper describes relationships among home and community environmental features, activity performance in the home, and community participation potential to support aging in place. A subset of data on older adults with functional limitations (*N* = 122), sixty three (63) with mobility and 59 with other limitations, were utilized in this study from a larger project's subject pool. Results showed significant and positive correlations between environmental barriers, activity dependence and difficulty at home, and less community participation in the mobility limitation group. While kitchen and bathroom features were most limiting to home performance, bathtub or shower was the only home feature, and destination social environment was the only community feature, that explained community participation. Compared to environmental features, home performance explained much more community participation. Study results provide detailed information about environmental features as well as types of home activities that can be prioritized as interventions for aging in place.

## 1. Introduction

Changes in the person-environment relationship as well as the negative outcomes of shrinkage in “life space” (i.e., the extent of mobility of older adults as measured by the range of places in which a person engages in activities within a designated time frame) associated with aging, particularly among seniors with mobility limitations, have been long conceptualized and widely documented [1–3]. In fact, restricted life space has been recently linked to increased risk of Alzheimer's disease [4]. Older adults have been reported to spend 80 percent of their time in their primary residence [5] and have demonstrated an “environmental centralization” of behaviors (i.e., the tendency of using a few preferred places at home where necessary or desired items are located) to maintain control and competence over the living environment [2, 6]. With almost 9 out of 10 (86%) older Americans reporting that they want to spend the rest of their lives in the homes and communities [7] in which the majority of their daily activities take place [8], a robust life space is essential for older adults to continue to engage and participate in as many home and community activities as independently and safely as possible. 

**Figure 1:**
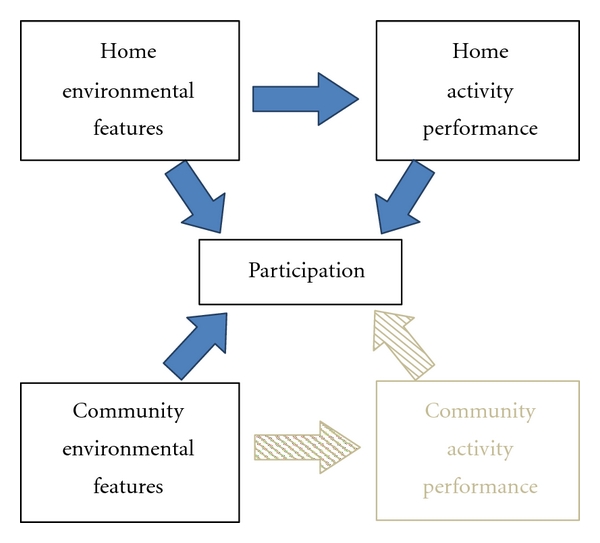
Based on the ICF, environmental press, and life space models, the conceptual framework for the study illustrates the relationship between home and community environmental features and home and community activity performance, respectively. In addition, activity as a prerequisite for participation suggests that home and community activity performance, in turn, influence participation in both settings although, as indicated by the dark filled arrows, this study only examined the relationships between community participation and (1) home and community environmental features and (2) home activity.

While prior work has consistently linked supportive home and community settings to continued performance of home activities and participation in community roles, respectively, evidence suggests that community participation, which is dependent on maintaining a wide range of life spaces outside the home, may also be affected by one's level of dependence and difficulty in performance of daily activities in the home [9]. Further, activity dependence and difficulty may be differentially affected by the same environmental features [10]. To develop a more comprehensive understanding of the factors that affect performance or activities in the home and participation in the community among older adults with limitations in mobility, this paper will describe the relationships among (1) home environmental features and performance of routine activities in the home as measured by task dependence and difficulty, (2) home and community environmental features and opportunities for community participation as measured by the frequency of travel to community life space destinations, such as restaurants, grocery stores, doctor's offices, and recreational areas, and (3) dependence and difficulty in home activities and opportunities for community participation (see Figure 1). 

### 1.1. Underlying Models

The theoretical bases of this study are derived from models of person-environment interaction, including two ecological models, the international classification of functioning, disability, and health (ICF) [11] and the environmental press model [1] and the life space model [12, 13]. The ICF provides a model that defines performance and participation as the interaction between the context, including the physical environment, and an individual's functional ability. The ICF also describes the interactions between activity performance and participation. Based on these underlying principles, this study examined the association between environmental factors and performance at home and participation in the community as well as interactions between home performance and community participation. 

The second ecological model, the environmental press model, has long played a major role in defining environmental contributions to activity and participation. Derived from the environmental press model, the environmental docility hypothesis suggests that the impact of demands is a function of an individual's ability. In other words, individuals with less ability will be impacted more by the same environmental demands than individuals with greater levels of ability. Based on the environmental docility hypothesis, this study examined the effects of environmental features on activity performance and community participation outcomes of older adults with and without mobility limitations. 

The life space questionnaire, as developed by Stalvey et al. [12], illustrates the movement trajectory of older adults in nine environmental zones from the bedroom, immediately outside the home (e.g., porch/patio), outside the home (e.g., yard or parking), immediate neighborhood, outside immediate neighborhood, outside the town, outside the county, outside the state, to outside the country. Due to the increased time spent inside home and decreased participation in the community among older adults [5], this paper used a simplified version of the life space concept that focuses on older adults' ability to move from inside their homes to life spaces in the community (including both immediate neighborhood and outside immediate neighborhood). As a result, use of community life space reflects the potential for community participation. This dichotomization from home to community spaces is critical as increasingly accessing community spaces provides and enhances opportunities for continued participation in societal roles [14].

### 1.2. Impact of Home and Community Environmental Factors on Activity and Participation

Most previous studies of environmental supports for aging in place have separately examined associations between either the home environment and activity performance or the community environments and participation. As a result, a comprehensive understanding of the interrelationships among home and community environments, performance, and participation is still lacking [15]. 

In *home settings,* unsupportive home features have been linked to greater difficulty and dependence in daily household activities [10, 16, 17]. In contrast, the provision of supportive environmental features, such as grab bars and home modifications, has been shown to enhance independence in activities, reduce caregiver burden, and decrease home care costs [18–20]. Most of these studies tend to associate the number of barriers/facilitators [20], a global score of environmental misfit [16, 17], or broad domains of home barriers/facilitators (e.g., overall bathroom barriers) [18, 19] to performance outcomes rather than linking specific home features (e.g., bathtub space or bathtub height) to specific performance outcomes (e.g., getting in and out of tub). As a result, these studies generally do not depict the differential impacts of specific home environmental features on explicit performance outcomes. One of the few exceptions [10] was a study that described correlations between home facilitators (i.e., home modifications) with both activity independence, and ease of performance in 15 home tasks by individuals with mobility impairments. However, none of the factors, home environmental features, activity independence or ease of performance were linked to community participation. 

In *public settings,* physical environmental factors, such as mixed land uses, highly connected street networks, availability (e.g., number and types) of stores and services, pedestrian-friendly streets and sidewalks, neighborhood attractiveness, and transportation, were linked not only to engagement in activity (e.g., exercise or walking to community destinations) of older adults, but also to their propensity to participate in society [21, 22]. Conversely, a study that examined the impact of specific environmental factors on activity and participation of seniors who used wheeled mobility aids reported that among the 50 factors examined, including 17 sidewalks, 17 crossings, 10 curb ramps, and 6 ramp characteristics, all 50 significantly prevented the 95 percentile of older wheelchair population from going out into the community, thus restricting opportunities for participation when those barriers were present [23]. Yet, other studies have found that the overall impact of the environment on participation was smaller than expected [24–27]. While mobility and balance (as opposed to other personal factors) explained 24% of participation in one study [24] and activity limitations explained much of community participation in another [25], in a third study, community environments, such as governmental and public services and physical environment and accessibility, only accounted for 6% of the variance in participation [26]. These data suggest that perhaps other factors, such as environmental factors in the home, may play a key role in community participation. 

In contrast to studies that focused on community features alone, Haak et al. [15] reported that a *continuum of home to community features* was significantly correlated with participation. However, while the study examined the impact of mostly social environmental supports in the community, such as good medical care in the vicinity, living close to friends and relatives, cultural opportunities in the vicinity, and having good local transport, specific physical environmental factors were not included. Moreover, although physical barriers in the home environments were included, these were aggregated to a number of environmental barriers and magnitude of accessibility problems. As a result, the impact of specific features on participation could not be assessed. 

To date, only one study has examined the interaction between home environmental factors and community participation. In a pre-post study prior to and after receiving home modifications for getting in and out of the house, moving around the house, and using the bathroom, Hammel et al. [9] examined older adults' ability to use a range of life spaces within and outside the home when they wanted and with whom they wanted. After receiving home modifications, participants not only reported an increased use of community life spaces, but they also used more distant life spaces. Most importantly, among all of the types of home modifications made, toilet and bathtub modifications, even more so than ramps and lifts for getting in and out of the house, demonstrated the largest effect on going out into the community. 

## 2. Purpose

Recognizing the complex interactions among environments, home activity performance, and community participation and the potential impacts on the ability of older Georgians to successfully age in place, the Georgia Council on Aging, which serves in an advisory capacity on aging issues to the Governor and General Assembly of Georgia, supported a survey to identify and prioritize the environmental and performance correlates of unmet home activity and community participation needs of Georgia's seniors. The goal of the survey was to develop a comprehensive understanding of home and community environmental barriers and facilitators that impact the activity and participation of Georgia's seniors to inform policy and prioritize service delivery needs for the State of Georgia. In addition, the data are useful in developing a more comprehensive understanding of community participation potential and life space restriction as a function of the interrelationships among home and community environmental features and home activity performance.

The data reported here described relationships between the home setting (i.e., environmental features and activity performance) and community participation potential of Georgia's seniors with mobility limitations compared to those without mobility limitations. Mobility limitation was selected as a subset of interest because this group is more likely to experience more environmental barriers and life space restriction than older adults with other limitations (i.e., hearing, vision, speech, and dexterity), but without mobility limitations. Specifically, the paper will address three key research questions by describing the associations among (1) home environmental features (i.e., barriers and facilitators) and activity performance in the home as measured by dependence and difficulty in home activities of seniors with mobility limitations compared to those with other limitations, (2) home and community environmental features and community participation potential as measured by usage of community life space (i.e., the frequency of going into community destinations, such as restaurants, grocery stores, doctor's offices, and recreational areas among older adults with mobility limitations compared those with other limitations) of seniors with mobility limitations compared to those with other limitations, and (3) home activity (i.e., dependence and difficulty) and community participation potential of seniors with mobility limitations compared to those with other limitations (see Table 1). 

## 3. Methods

The study employed a cross-sectional survey design to explore the relationships among environmental features, dependence and difficulty in activity performance in the home, and life space usage in the community participation to understand the met and unmet activity and participation needs of older Georgians. A web-based survey hosted by Survey Gizmo was developed to solicit input from Georgia's seniors. Survey Gizmo was chosen because it is compliant with Section 508 of the Rehabilitation Act and is generally the most accessible and usable online survey platform. In addition, it utilizes an encrypted connection to ensure confidentiality of data. To ensure inclusion of older adults who did not have access to an online platform, alternative paper and telephone formats were made available. This project was approved by the Georgia Tech Institutional Review Board (IRB).

### 3.1. Participants

A total of 239 individuals with and without functional limitations who were 60+, living in the State of Georgia and had resided in their current residence for at least one year, were recruited for the survey. Seven out of 179 online surveys and 6 out of 54 written surveys were eliminated due to large amounts of missing data, resulting in a total of 226 participants in the study. 

Participants were asked to indicate whether they experienced one or more of five functional limitations, including vision, hearing, speaking, moving around, and hand manipulation. Among the total of 226 participants, 122 had at least one of the five functional limitations. The 63 respondents who answered “yes” to the question: “do you have difficulty with moving around?” were included in the mobility limitation (ML) subset reported in this paper. The 59 participants who responded with a “yes” to any of the other 4 limitations were included in the other limitation (OL) group. It should be noted that because participants could respond to more than one limitation, the OL group included 52 individuals who had vision, 60 who had hearing, 26 who had hand manipulation, and 4 who had speaking limitations.

### 3.2. Procedures

A convenience sampling technique was employed in order to reach the required sample of 200 participants to achieve a statistical power of 80. Subject recruitment took place from November, 2009 to September, 2010. Participants were recruited through a variety of methods, including subject registries maintained by the Center for Assistive Technology and Environmental Access at Georgia Tech, as well as through email invitations and posts at AAAs, AARP, NORCs, senior centers, and other senior-related organizations throughout Georgia. Both email invitations and posts were provided with a brief study description and researchers' contact information so that potential subjects who were interested in the study could actively contact the researchers by phone or email. All subjects that expressed an interest in the study were given a more detailed study description and a written informed consent form. Those who consented to participate were given the choice of taking an on-line, written, or telephone survey. Those who chose an on-line method (*n* = 179) were emailed with the link to the on-line survey. Hardcopy, text versions of the survey (*n* = 54) were mailed out with a self-addressed stamped envelope. The telephone survey (*n* = 6) was scheduled with the participant at a time that was mutually agreeable. The survey took approximately 20 minutes online and 30–40 minutes in a written or telephone format. 

### 3.3. Survey Instrument

The survey gathered self-perceive information on a variety of factors, including (1) activity performance, (2) community participation potential, (3) environmental barriers and facilitators, and (4) participant demographics. All survey questions were answered by participants without assistance from the researchers. Based on a comprehensive review of literature, the survey borrowed from and adapted questions from a number of existing instruments, including *comprehensive assessment and solution process for aging residents (CASPAR) [28], the healthy aging research network (HAN) environmental audit tool and protocol [29], the facilitators and barriers survey of environmental influences on participation among people with lower limb mobility impairments and limitations* (FABS/M) [30], and the *participation survey: mobility* (PARTS/M) [31].

Activity performance and environmental barriers/facilitators in the home were adapted from the CASPAR [28]. CASPAR was chosen because it associates demand-producing environmental attributes (which could be barriers or facilitators) with actual activity performance [28, 32]. In contrast, other existing home assessment instruments that compare environmental attributes to performance, such as the housing enabler [33], focus on environmental barriers and not assess actual performance. Rather, activity performance (e.g., cannot go up and down the ramp to get in and out of the house) is predicted from a comparison of environmental attributes that are expected to be barriers (e.g., ramp slope) to an individual with functional limitations that interact with those attributes (e.g., lower body motor limitation). This approach is useful in informing decisions about home modification needs when actual performance cannot be observed, such as prior to an individual's discharge from a rehabilitation facility, but because it only predicts performance that may or may not actually occur, it may result in false positives as well as underestimating problems. As a result, these types of predictive assessments did not provide sufficient information to make decisions about environmental modification needs. CASPAR, in contrast, which associates environmental attributes against actual activity performance, results in information that could be used by the Georgia Council on Aging to determine actual environmental modification needs [34]. 

The CASPAR includes self-reported information on functional abilities, types of performance problems with person-environmental transactions, such as getting on and off toilet, and detailed measures of activity-relevant environmental attributes of the home, such as height and location of toilet. The sections of performance and environmental attributes were utilized and adapted for the current study. For example, in the environmental section, direct measurements of home attributes such as bathtub dimensions were omitted as provision of modification intervention was not the intent of this study. Instead, perceived impacts of environmental attributes on matching task performance were surveyed. That is, participants were asked to rate on a 5-point Likert scale from “limits a lot” to “helps a lot” to be consistent with the response system in the measure of community environment. In addition, to shorten the time required for survey administration, the number of home environmental features in CASPAR was reduced from features in eight activity areas (i.e., getting in and out of the house, using interior stairs, moving around the house, using the bathroom, using the bedroom, using the kitchen, using the laundry, and controlling ambient conditions) to those in four activity areas that were considered to be the most crucial for daily home activities (i.e., getting into and around the home, using the bathroom, using the kitchen, and using the bedroom). Interrater reliability and criterion validity of CASPAR were moderate to high on the majority of items [28]. 

Measures of community environmental barriers/facilitators were adapted from the *healthy aging research network (HAN) environmental audit tool and protocol *[29] and the* facilitators and barriers survey (FABS/M)* of environmental influences on participation among people with lower limb mobility impairments and limitations [30]. The HAN environmental audit tool and protocol was designed for research purposes and developed through both qualitative interviews and quantitative reliability testing at multiple sites [29]. This tool was chosen because it covers both physical and social attributes in the community. The total number of attributes in the original tool was 55, and they were grouped by the study researchers into a final list of 7 community features (i.e., stores, streets, sidewalks, visual appeal, public transit, and destination physical and social support) with descriptions of the original attributes under each of the seven categories in order to reduce survey burden placed on the participants. In addition, because the HAN environmental audit tool used both ordinal and categorical data, it did not lend itself to the ordinal scoring system needed to measure the magnitude of environmental features as barriers or facilitators to performance [29]. As a result, the ordinal response options in the FABS/M were adopted to measure person-environmental transactions (i.e., impact of community features and attributes on corresponding community activities or behaviors). The response options utilized a 5-point Likert scale from “limits a lot” to “helps a lot.” The FABS/M is a widely used measurement on community environments with sound psychometric properties [30]; however, as the FABS/M was not originally developed for the older population, its survey questions could not fully capture barriers and facilitators encountered by older adults. Therefore, only the response system was utilized in our study. 

Finally, the *participation survey: mobility (PARTS/M)* was used to develop measures of community participation potential [31]. The PARTS/M was developed based on the international classification of functioning, disability, and health (ICF) and had good internal consistency and stability [31]. The PARTS/M measures the frequency of traveling to various community settings (e.g., restaurants, bank, doctor, and grocery) as an indicator of the potential for participation. In other words, travel to community destinations is a perquisite to participation in societal roles. The more frequently individuals traveled to community destinations, the greater the likelihood that they would participate in societal roles. Conversely, the less often they traveled to community destinations, the fewer opportunities they would have to participate in societal roles. 

### 3.4. Independent Measures

Independent variables included both *environmental features* rated as either barriers or facilitators and *functional limitations*. *Environmental features* included 17 features (e.g., steps, toilets, kitchen appliances, and bedroom closets) in four areas of the home (i.e., circulation, bathroom, kitchen, and bedroom) and 7 features in the community (i.e., stores, streets, sidewalks, visual appeal, public transit, and destination physical and social support). The degree to which any environmental feature was perceived to be a barrier or facilitator was defined by the perceived level of support, on a 5-point Likert scale from 1 = “helps a lot” to 5 = “limits a lot,” that was afforded by any particular feature. 

*Functional limitations* were divided into two groups: mobility limitation group (ML) as defined by difficulty moving around and other limitations group (OL), as defined by having difficulty with vision, hearing, speaking, and/or hand manipulation, were used as the other independent variable. Each of the limitations was measured dichotomously (i.e., with or without a specific functional limitation). Respondents could select more than one limitation if applicable. 

### 3.5. Outcome Measures

Dependent outcome measures included *activity performance at home* and *participation potential in the community. Activity performance *was measured by activity independence/dependence as well as ease/difficulty. Activity independence/dependence was defined as needing personal assistance while performing an activity, regardless of the use of assistive technology. Activity dependence was reported by subjects on a 3-point Likert scale from 1 = “independent,” 2 = “dependent,” to 3 = “unable to perform the activity.” Activity ease/difficulty was defined as self-reported ease or difficulty in performing each activity in the usual way (i.e., with or without assistance of another person). Four levels of perceived difficulty from 1 = “no difficulty,” 2 = “somewhat difficult,” 3 = “very difficult,” to 4 = “unable to perform the activity” were assessed. Both activity independence and difficulty address routine performance, that is, actual performance rather capacity to perform. The activities queried in the survey were adapted from the CASPAR, including three *circulation* tasks (getting in and out of the house, going up and down interior stairs, and moving around inside the house), two tasks for using the *bathroom *(getting on and off a toilet, getting in and out of a bathtub or shower), three for using the *kitchen* (using kitchen appliances, getting items in and out of upper cabinets, and getting items in and out of lower cabinets), and the two for using the *bedroom *(getting on and off a bed and using the closet). 

*Participation potential* [35], adopted from the PARTS/M, was defined as self-reported frequency of actual community participation. It was assessed by one question, “how often do you actually go into destinations (such as restaurants, banks, churches, and recreational areas) in your community ?” on six levels of frequency from “daily,” “several times a week,” “several times a month,” “once a month,” “less than once a month,” and “do not participate in the community.” 

### 3.6. Demographic Data

Demographic data were used to describe the study sample, including *age* (i.e., year born), *gender*, *ethnicity* (i.e., white/Caucasian, African American, Hispanic or Latino, Asian, Native American/Alaskan Native, Native Hawaiian/other Pacific Islander, and other), *education levels* (i.e., no high school, some high school, high school diploma/GED, associate degree, bachelor's degree, master's degree, and doctorate degree), *community types* (i.e., urban, suburban, and rural areas), *mobility aids* (i.e., cane, crutch, walker, manual wheelchair, power wheelchair, and scooter) *and sensory devices* (i.e., hearing aids and glasses).

### 3.7. Data Analysis

Data from the online survey (*n* = 172) were automatically entered into an online database. Data from the written (*n* = 48) and telephone survey (*n* = 6) were hand entered. All written and telephone survey data were double entered to ensure accuracy. Spearman rho correlations were conducted for all three research questions, that is, to associate (1) home environmental features to independence and to difficulty of home activities, (2) home and community environmental features to participation potential, and (3) independence and difficulty of home activities to participation potential. Since matched sets of activity and activity-related environmental features (e.g., getting on/off toilet and toilet space and toilet) were used in research question 1, stepwise regressions were only conducted for research questions 2 and 3. Four stepwise regressions were further conducted to individually identify which (1) home and (2) community environmental feature that explain community participation for research question 2, and (3) independence and (4) difficulty in which home activities explain community participation for research question 3. Cohen effect size conventions of small = 0.10, medium = 0.30, and large = 0.50 were used for both correlation and regression analyses [36]. Both moderate and large effect sizes are interpreted to be clinically significant. Descriptive comparisons of all independent and outcome variables between the mobility and other limitation groups were all conducted by Chi-square analyses. Due to an exploratory and descriptive nature of this paper, results were considered to be significant at *P* < .05. Because the analysis of this data employed multiple independent analyses, uncorrected significance tests are not appropriate for inferential interpretation. However, significance is reported here with uncorrected *P* values to be interpreted as an arbitrary criterion of effect size strength in deference to its widespread use in social science for exploratory analyses.

## 4. Results

### 4.1. Descriptive Analyses

#### 4.1.1. Sample Demographics

A total of 122 participants met the criteria of having “functional limitations” and were included in this analysis. Among these, the sample was approximately equally divided between the ML (*n* = 63, 51.6%) and OL groups (*n* = 59, 48.4%). Overall, the ML was fairly high functioning. More than 4 out of 10 (41%) did not use mobility aids, while an almost equivalent percentage (38%) used a cane. Only 18% used walkers, 8% used power wheelchairs, 3% used manual wheelchairs, and 2% each used crutches and scooters. Almost two-thirds (64%) of the OL group had a hearing limitation (see Table 2). 

The mean age of all participants with functional limitations was 72.5 (S.D. = 8.50) with the ML group being 71.2 years of age and the OL group being slightly older at 73.9 years of age, although the difference was not significant. The majority of the respondents was female (64%), Caucasian (74%), and living in suburban (51%) areas (see Table 2). In addition, almost one-third (32%) had an associate or bachelor degree. There were no significant differences in race or residence between the ML and OL groups (73% and 74% Caucasian; 50% and 51% living in suburban areas, resp.) although there were significant differences (*P* < .01) in gender (73% female in the ML group versus 54% in the OL group). 

#### 4.1.2. Environmental Features as Barriers and Facilitators

Overall, only approximately one in five respondents perceived barriers in either the home (*n* = 18, 14.8%) or in the community (*n* = 26, 21.3%). The most common home barriers reported by the whole sample included kitchen cabinets (24.8%), bathtubs or showers (23.5%), bedroom closets (23.5%), and steps (19.2%). The most common community barriers were streets (28.0%), sidewalks (28.0%), and number and of stores (23.5%). Conversely, the bathroom sink was the feature perceived by the lowest percentage (8.4%) of the whole sample in the home, whereas social environments in community destinations were perceived by the lowest percentage of respondents (14.4%) as a barrier in the community. 

Similar to the whole sample, both ML and OL groups perceived more barriers in the community than in the home. Although the ML group perceived more barriers in each of the settings with 29% perceiving community barriers to 18% in the OL group and 17% perceiving home barriers to 12% in the OL group, neither was statistically significant. The home and community barriers cited most often by the largest percentage of the ML and OL groups were also similar to the whole sample. However, all home and community features were perceived as barriers by higher percentages in the ML group than the OL group with the exception of public transportation, which had equal percentages in both groups. However, among these features, only steps (*P* < .05) and kitchen cabinets (*P* < .05) in the home and the physical environment in community destinations (*P* < .01) were significantly higher in the ML than the OL group (see Table 3). 

#### 4.1.3. Activity Performance: Dependence and Difficulty

Dependence in each of the ten home activities ranged from 4.3% to 33.6% for the overall sample, with the largest percentage of respondents (33.6%) being dependent in getting items in and out of upper cabinets in the kitchen and the smallest percentage of respondents being dependent in getting on and off a toilet (4.3%). A significantly higher percentage of the ML group reported being more dependent than the OL group in eight of the ten home activities (*P* = .000–.038). Moreover, the trend continued with a higher frequency of respondents in the ML group reporting greater dependence in the other two activities, moving around inside house and getting items in and out of a closet, although the differences between groups were not significant.

Compared to activity dependence, higher percentages of the overall study sample reported having difficulty with the 10 activities ranging from 13% to 53.0%. The largest percentage of respondents had difficulty going up and down stairs (53%), whereas the smallest percentage had difficulty getting on and off a bed (13%). Similar to activity dependence, higher percentages of the ML group reported having difficulty in all ten home activities, although in this case, all activities were significantly (*P* = .000–.022) more difficult in the ML than the OL group (see Table 4). 

#### 4.1.4. Community Participation Potential: Frequency of Use of Community Life Spaces

In general, older adults in the study sample were generally active. Almost three-quarters (*n* = 88, 72.5%) of the overall study sample went into community at least several times a week (*n* = 48, 39.7%) or everyday (*n* = 40, 32.8%). Despite the large number of participants who were active, more than one-quarter demonstrated restricted life space by traveling to community destinations less than weekly (18.1%, *n* = 22) or once a month or less (9.5%, *n* = 12). 

When the ML and OL groups were compared, as expected, a lower percentage of the ML group participated in the community everyday (28.6%) compared to the OL group (37.7%), although the differences were not statistically significant. The differences in community participation between the ML and OL groups were most evident among the least active community participants, with a trend (*P* = .054) toward more participants in the ML group (14.3%) demonstrating life space restriction (i.e., going into community every month or less) than the OL group (3.8%). 

### 4.2. Correlational Analyses

**RQ1: What Is the Relationship between Home Environmental Features and Activity Performance in the Home?**

While almost none of the home features were significantly correlated with activity performance in the OL group, over half of the home barriers were significantly correlated with either activity dependence or difficulty in the ML group. More than three-quarters (76.6%) of the 17 home barriers were significantly correlated with activity difficulty, while 58.8% (*n* = 10) were significantly correlated with dependence.

Among the features in the four home spaces included in the study, all features in the kitchen, including kitchen space, appliances, and cabinets, positively (*r* = 268–.627) and significantly (*P* < .001–<.05) correlated with both difficulty and dependence in performing the corresponding activities (i.e., using kitchen appliances, and getting items in/out of upper cabinets, getting items in/out of lower drawers). Two of the circulation features, steps and going up and down stairs, were significantly correlated with both the dependence and difficulty in going up and down stairs and moving around the house, respectively. Steps were positively and significantly correlated to dependence (*r* = .520; *P* < .001), and difficulty (*r* = .303; *P* < .05) in going up and down stairs, whereas home space barriers were positively and significantly correlated with dependence (*r* = .377, *P* < .01) and difficulty (*r* = .364, *P* < .01) in moving around the house. The other four circulation features were correlated with either dependence (i.e., walkway and doorway) or difficulty (i.e., pathway and door) with *r* values ranging from *r* = .276–.434 and significance ranging from *P* < .001 to *P* < .05. In the bathroom, toilet features barriers were significantly correlated with both dependence (*r* = .327; *P* < .01) and difficulty (*r* = .268; *P* < .05) in getting on and off toilet; however, tub/shower features were only significantly correlated (*r* = .257; *P* < .05) with difficulty in getting in and out of bathtub/shower. Interestingly, neither space at the toilet nor at the tub/shower was significantly correlated with dependence or difficulty in getting on or off the toilet or in and out of the shower. Finally, in the bedroom, closet features were significantly (*r* = .503; *P* < .001) correlated with both dependence and difficulty in getting items in and out of a bedroom closet. The other two bedroom features, bedroom space (*r* = .393, *P* < .01) and bed (*r* = .468, *P* < .001), were significantly correlated with activity difficulty (see Table 5). 

In the OL group, only three home features, kitchen cabinets, bed, and steps, were significantly correlated with activity performance at home. Two features, kitchen cabinets and bed, were positively correlated with difficulty in getting items in and out of lower drawers (*r* = .292; *P* < .05) and difficulty getting out of bed (*r* = .316; *P* < .05), respectively. In contrast, two home features, kitchen cabinets and steps, were negatively correlated with activity. Kitchen cabinets were significantly correlated (*r* = −.342; *P* < .05) with getting items in and out of upper cabinets, whereas steps were significantly correlated (*r* = −.355; *P* < .05) with independence in going up and down stairs.

**RQ2:What Is the Relationship between Home and Community Environmental Features and Potential for Community Participation?**

Whereas community environmental features were significantly correlated to frequency of travel to community destinations in the ML group, they were not significantly correlated in the OL group. Although no environmental features in the home were significantly correlated with frequency of travel to community destinations in either the ML or OL groups, among the 7 community features, three, including streets (*r* = .294; *P* < .05), sidewalks (*r* = .283; *P* < .05), and social environments of community destinations (*r* = .346; *P* < .01), were significantly correlated with frequency of going into community in the ML group.

Stepwise regressions were undertaken to further identify home and community features that explained travel frequency among the ML group. Results indicate that bathtub/shower was the only home feature that explains any significant amount, although slightly less than 6% (adjusted *r* ^2^ = .055; *P* < .05) of travel frequency. In the community, social environments at a destination was the only feature that accounts for a significant amount of variance (adjusted *r* ^2^ = .130; *P* < .01).

However, when frequency of travel to community destinations is dichotomized into frequent travelers (i.e., “more than once a month”) and infrequent travelers (“once a month and less”), frequency was significantly correlated with the majority of both home (64.7%, *n* = 11) and community (71.4%, *n* = 5) features in the ML group. Among home features, kitchen and bathroom features had the highest percentage of features that were significantly correlated with infrequency of travel to community destinations in the ML group, including four out of five (80.0%) bathroom features (*r* = .289–.401; *P* < .01 to *P* < .05) and all three (100.0%) kitchen features (*r* = .252–.301, *P* < .05). Among community features, stores, sidewalks, visual appeal, physical environments, and social environments were significantly correlated (*r* = .268–.431, *P* < .001 to *P* < .05) with infrequent travel in the ML group (see Table 6). In contrast, no home or community features were significantly correlated with dichotomized frequency of travel in the OL group.

In addition to correlations between environmental features and dichotomized participation, odds ratios were calculated for dichotomized environmental features (i.e., barriers/facilitators) and dichotomized participation (i.e., frequent/infrequent travel). In the home setting, all bathroom features, with the exception of bathroom sinks, had significant odds ratio results (i.e., the upper and lower CI95% did not overlap 1.00) in the ML group, while there were no significant results in the OL group. When toilet space, toilet, tub/shower space, and tub/shower were perceived as barriers, the odds of infrequent travel were 46.7, 25.0, 29.0, and 8.0 times higher, respectively, compared to when they were perceived as facilitators. Among community features, the odds of infrequent community travel were 17.8 times higher in the ML group when sidewalks were perceived as barriers and 21.3 times higher when social environments at the destination were perceived as barriers. Similarly, there were no significant odds ratios in the OL group in community settings.

**RQ3: What Is the Relationship between Activity Performance in the Home and Community Participation Potential?**

As in other analyses, significant correlations were only found in the ML group. Again, in the ML group, there were a greater number of significant correlations between frequency of travel to community destinations and both kitchen and bathroom activities than circulation activities. There were no significant correlations between bedroom activities and frequency of travel to community destinations (see Table 7).

Comparing kitchen and bathroom activities, dependence of all kitchen activities and difficulty in all bathroom activities were positively correlated with less community participation. In the kitchen, dependence in using kitchen appliances, getting items in and out of upper cabinets and of lower drawers were significantly correlated with less community participation (*r* = .272–.417; *P* < .01 to *P* < .05). Among these three activities, getting items in and out of upper cabinets showed the strongest correlation (*r* = .417). In the bathroom, difficulty in getting on and off a toilet and getting in and out of a bathtub/shower were significantly correlated with less frequent travel to community destinations (*r* = .259 and .438, *P* < .05 and *P* < .001, resp.). In circulation-related activities, both dependence in getting in and out of the house and going up and down stairs, as well as difficulty in getting in and out of the house, is significantly correlated (*r* = .406–.463; *P* < .001 to *P* < .01) with less frequent travel to community destinations. 

To identify the home activities that account for the largest variance in frequency of travel to community destinations, two stepwise regressions were undertaken. Activity dependence and difficulty were entered separately. For activity dependence, getting items in and out of upper cabinets and getting in and out of house explained approximately one-fourth (adjusted *r* ^2^ = .246; *P* < .001) of the variance in travel frequency. For activity difficulty, getting in and out of a bathtub/shower is the only significant activity in the model, accounting for almost one-third of frequency of going into community (adjusted *r* ^2^ = .  306; *P* < .001). 

## 5. Discussion

This study described relationships among home and community environmental features, dependence and difficulty in activity performance at home, and frequency of travel to community life space as an indicator of participation potential. Specifically, the study demonstrated that home environmental features were not only significantly associated with activity difficulty and dependence in the home, but also with less restriction in life space. In fact, the latter was positively related to home features and home performance as well as community environmental features. These results clearly demonstrated our primary hypothesis that remotely located home environmental features and activity performance can impact community participation. 

As expected, correlations were only significant among the ML group. However, this group also had lower performance and participation outcomes, which supports findings by Anaby and colleagues [24] that mobility and balance, more so than any other limitations, account for the largest variance in performance and participation. The lower performance and participation outcomes as well as the stronger link between environment, performance, and participation not only indicate the higher vulnerability in people with mobility limitations to age in place, but also postulate that both performance and environmental intervention are a potentially important strategies to facilitate aging in place.

**RQ1: Home Features and Activity Performance at Home**

Previous studies have identified top barriers to activity performance at home as well as environmental features to reduce dependence and difficulty [10, 16–20]. This study not only provides further evidence that home features impact difficulty and dependence among mobility impaired seniors, but also suggests that features can be either barriers or facilitators. Kitchen features presented the primary barriers to both dependence and activity at home. This is not surprising as research and practice suggest that kitchen features are low-priority modifications as they are expensive, and kitchen activities are perceived to be easily substituted or skipped without impacting daily functions greatly, particularly in comparison to more critical bathroom and entry/exit modifications. As a result, the data suggest that many of the participants had modification needs in the kitchen. Surprisingly, bathroom features (i.e., tubs, showers, and toilets), rather than space, were significantly correlated to activity performance, which perhaps reflects the vast majority (almost 90%) of the sample that was ambulatory. Since maneuvering space is a factor that primarily affects wheelchair users, the results may be different if the sample had a larger number of wheelchair users.

**RQ2: Environmental Features and Community Participation Potential**

Not surprisingly, community features were more strongly related with overall community travel than home features. Nonetheless, like their impact on home activity, the majority of bathroom and kitchen features were also significantly related to infrequent community travel. This was particularly true of bathtub or shower design (e.g., size of bathtub or shower, height of bathtub edge, or shower threshold) which accounted for a significant amount of the variance in travel frequency. Moreover, when the four toilet and bathing features, toilet space, toilet design, tub/shower space, and tub/shower design, were perceived as barriers, respondents were 8 to 46.7 times more likely to travel into the community only once a month or less.

These findings are consistent with a previous study [9], which reported large effect sizes of toilet and bathing interventions on community travel. One possible explanation is the toilet and bathtub create such significant barriers such that the amount of time and energy required to toilet and bathe limits the amount of time that can be spent in the community. Alternatively, people may feel that the barriers limit their personal hygiene activities and are therefore less willing to travel into the community. 

In addition to home environmental features, the social environment at community destinations, such as staff's willingness to offer assistance in a restaurant, not only showed the strongest correlation among all community features, but also it was the only community feature that attained significance in explaining the variance in community travel frequency. Social environment at community destinations also shows stronger odds ratio to community participation between the only two significant community features. However, together bathtub/shower and destination social environments only explained little (5.5% to13%) of community travel. Such findings are consistent with previous literature, in which community accessibility only accounted for 6% of the variances in participation [26]. Our finding of positive correlations between home barriers and less community participation was also consistent with results from Haak and colleagues [15] that significant correlations between the number of home barriers and community participation were reported. Despite this, our findings identified specific home and community features that were significantly correlated to participation, which suggests a potential direction for further research, if not environmental intervention.

**RQ3: Activity Performance at Home and Community Participation Potential**

Similar to home barriers, performance challenges at home were positively correlated to less community participation, especially in kitchen, bathroom, and circulation-related activities. Dependence in getting items in and out of upper kitchen cabinets and getting in and out of the house explained 24.6% of community travel patterns. The former was significantly related to barriers of upper cabinets in the kitchen, including height of cabinets and handle styles, and it entails the likelihood of a positive association between decreased mobility/balance function and increased difficulty in community participation. The latter was significantly related to barriers of walkways such as physical condition or material of the street, driveway, and lighting, and it points out the importance of achieving independence in getting in and out of the house because assistance cannot be always handy.

In addition to dependence at home, difficulty in getting in and out of bathtub or shower also explained 30.5% of community travel. One possible explanation is the increased time and energy spent in the bathroom that reduces the time and energy available for going out into the community. Difficulty in getting in and out of the tub or shower was significantly correlated to barriers in the tub or shower, which also corresponds to predictors of home barriers to community participation. While previous research has shown the strong contribution of activity limitation to participation [25], our findings further described the type of home activity as well as the kind of performance indicator related to community participation. 

This study provides the first step to comprehensively understand the relationships between home and community environments, home performance, and community participation as they impact aging in place. However, the study was limited by a number of factors including a small sample of convenience, which resulted in small effect sizes on many correlation results, and, ultimately limited generalizability of the data. The sample itself was an artifact of the time frame and funding allocated to the project, which limited the sample size and the data collection options. Although many of the correlations in the results had small to medium effect sizes, the prediction of difficulty in using the tub/shower to community participation achieved a moderate to large effect size. Both moderate and large effect sizes are interpreted to be clinically significant [36]. Future studies should increase the sample size to enhance the effect size. Further studies should also include and control for covariates, such as functional level and living situation, in the examination of the environment and performance predictors for aging in place. However, despite the limitations, to the authors' knowledge, this is one of the first studies that provides a more robust and comprehensive understanding of the impact of home and community environmental factors on home activity performance as well as community participation of older adults. Such an understanding of the impact of home and community features as well as itemized home activities provides a more viable “recipe” for intervention to facilitate aging in place.

## 6. Implications

This study has several implications for policy makers and practitioners. First, individuals with mobility limitations were found to be more vulnerable to the environment than those with other types of limitations, which suggests that environmental interventions for aging in place should first target at older adults who have difficulty moving around as they are the most environmentally vulnerable. Most importantly, environmental modifications should be conceived as a continuum of interventions from home to community in order to support both the activities and community participation that are necessary for successfully aging in place. In doing so, understanding the effects of interventions across settings is an important tool in identifying and prioritizing environmental modification needs for making decisions in policy and practice. 

In addition, findings from this study suggest that contrary to current policy and practice that focus on independence as the primary intervention goal, both dependence and difficulty in activity performance predicted much of community participation. In fact, more home barriers were correlated with difficulty than dependence at home in our sample. Many older adults may not ask for assistance at the onset of functional declinations but may have already started experiencing difficulty in basic home activities. This may not only reduce the time they have available for community participation and other meaningful activities but may also pose potential safety hazards. Therefore, reducing activity difficulty should be a directed intervention goal in order to detect early unmet needs for aging in place. 

Finally, our results provide more detailed information about environmental features that can be prioritized as interventions for aging in place. Targeted home features to enhance both home performance and community included accessible bathtubs or shower, walkways, and kitchen features. In the community, it is important to pay more attention to the social environment in the destinations to promote participation. Ultimately, a good physical environment will never overcome a bad social environment, but a good social environment can overcome a bad physical environment. Possible interventions could include disability awareness training for all community members and community social support system and network, to enable older adults to participate in the community and successfully age in place.

## Figures and Tables

**Table 1:** Research questions.

		Dependent variables	
		Home activities	Community participation
Independent variables	*Home features*	RQ1	RQ2
	*Community features*		RQ2
	*Home activities*		RQ3

**Table 2:** Demographics.

	All (*N* = 122)	Mobility/other limitation (*n* = 63)/(*n* = 59)
Age		
Mean (S.D.)	72.5 (8.50)	71.2 (8.30)/73.9 (8.58)
Gender		
Male	36%	27%/46%
Female	64%	73%/54%*
Ethnicity		
Caucasian	74%	73%/74%
African American	22%	21%/22%
Hispanic/Latino	1%	2%/2%
Native American	2%	0%/2%
Other	3%	5%/0%
Education		
No/some high school	12%	14%/9%
High school/GED	30%	35%/25%
Associate/bachelors	32%	30%/33%
Graduate degrees	26%	21%/33%
Community types		
Urban	20%	21%/19%
Suburban	51%	50%/51%
Rural	29%	29%/27%
Functional limitations		
Difficulty with seeing	43%	40%/46%
Difficulty with hearing	49%	35%/64%^†^
Difficulty with speaking	3%	5%/1%
Difficulty with moving around	48%	100%/0%
Difficulty with manipulation	21%	18%/8%*

*P < .05; ^†^ *P* < .01.

**Table 3:** Perceived home and community barriers by percentage of responses.

	Mobility/other limitation
Features	Barriers (%)^1^
Circulation	
Walkway	19.4/8.5
Steps	24.2/13.8*
Doorway	12.9/7.0
Home space	16.9/13.8
Pathways	15.0/6.8
Door	13.6/10.0
Bathroom	
Toilet space	11.3/7.1
Toilet	16.1/12.3
Tub/shower space	14.5/10.5
Tub/shower	22.6/19.0
Sink	11.3/5.3
Kitchen	
Space	16.1/16.9
Appliances	11.5/11.9
Cabinets	30.6/18.6*
Bedroom	
Space	12.9/8.5
Bed	17.7/12.1
Closet	29.5/17.2
Community	
Stores	27.9/19.0
Streets	29.0/27.1
Sidewalks	37.1/24.1
Visual appeal	16.7/13.6
Public transit	17.2/17.2
Dest. physical	34.9/13.5^†^
Dest. social	27.1/11.1

^1^Percentage of responses on “limit some” and “limit a lot.”

*P < .05; ^†^ *P* < .01.

**Table 4:** Comparisons of performance and participation between mobility and other limitation groups.

	Mobility/other limitation	
Performance	Dependence (%)^1^	Difficulty (%)^2^
Circulation		
Getting in and out of the house	15.9/0.0^†^	55.6/7.5^‡^
Going up and down stairs	39.3/9.3^†^	82.5/18.5^‡^
Moving around inside house	9.5/1.9	28.6/3.7^†^
Bathroom		
Getting on and off a toilet	7.9/0.0*	25.8/5.6*
Getting in and out of a bathtub or shower	14.3/0.0^†^	48.3/7.4^‡^
Kitchen		
Using kitchen appliances	17.5/1.9*	33.9/7.4^†^
Getting items in and out of upper cabinets	51.6/13.0^‡^	64.5/32.1^†^
Getting items in and out of lower drawers	27.4/1.9^†^	54.8/7.4^‡^
Bedroom		
Getting on and off a bed	9.7/0.0*	21.3/3.7*
Getting items in and out of a closet	6.1/3.7	24.6/5.7*

Participation	Frequency	Monthly^3^
	*P* value	*P* value

Community		
Going into your community	N.S.	.054

^1^Percentage of responses on “dependent” and “unable to perform.”

^2^Percentage of responses on “somewhat difficult,” “very difficulty,” and “unable to perform.”

^3^Going into community ≤ or > once per month.

*P < .05; ^†^ *P* < .01; ^‡^ *P* < .001.

**Table 5:** Correlations between environmental features and (1) activity independence-dependence and (2) activity ease-difficulty.

	Mobility limitation	
Features	Independence-dependence	Ease-difficulty
Circulation		
	Getting in/out of the house	Getting in/out of the house
Walkway	.254*	N.S.
Steps	N.S.	N.S.
	Going up/down stairs	Going up/down stairs
Steps	.520^‡^	.303*
	Moving around the house	Moving around the house
Doorway	.434^‡^	N.S.
Home space	.377^†^	.364^†^
Pathways	N.S.	.276*
Door	N.S.	.297*
Bathroom		
	Getting on/off toilet	Getting on/off toilet
Toilet space	N.S.	N.S.
Toilet	.327^†^	.268*
	Getting in/out bathtub	Getting in/out bathtub
Tub/shower space	N.S.	N.S.
Tub/shower	N.S.	.257*
Kitchen		
	Using kitchen appliances	Using kitchen appliances
Space	.461^‡^	.391^†^
Appliances	.467^‡^	.443^‡^
	Getting items in/out of upper cabinets	Getting items in/out of upper cabinets
Cabinets	.268*	.269*
	Getting items in/out of lower drawers	Getting items in/out of lower drawers
Cabinets	.464^‡^	.627^‡^
Bedroom		
	Getting on/off a bed	Getting on/off a bed
Space	N.S.	.468^‡^
Bed	N.S.	.393^†^
	Getting items in/out of a closet	Getting items in/out of a closet
Closet	.503^‡^	.570^‡^

*P < .05; ^†^ *P* < .01; ^‡^ *P* < .001.

**Table 6:** Correlations between environmental features and community participation and odds ratio results.

	Mobility limitation	
Features	Going into community ≤ or > once per month	
	*r*	OR
Circulation		
Walkway	.314*	N.S.
Steps	N.S.	N.S.
Doorway	N.S.	N.S.
Home space	.284*	N.S.
Pathways	N.S.	N.S.
Door	N.S.	N.S.
Bathroom		
Toilet space	.402^†^	46.7
Toilet	.357^†^	25.0
Tub/shower space	.376^†^	29.0
Tub/shower	.289*	8.0
Sink	N.S.	N.S.
Kitchen		
Space	.301*	N.S.
Appliances	.278*	N.S.
Cabinets	.252*	N.S.
Bedroom		
Space	.355^†^	N.S.
Bed	.254*	N.S.
Closet	N.S.	N.S.
Community		
Stores	.286*	N.S.
Streets	N.S.	N.S.
Sidewalks	.268*	17.8
Visual appeal	.297*	N.S.
Public transit	N.S.	N.S.
Dest. physical	.276*	N.S.
Dest. social	.431^‡^	21.3

*P < .05; ^†^ *P* < .01; ^‡^ *P* < .001 (Spearman's rho).

**Table 7:** Correlations between activity performance at home and community participation.

	Mobility disability	
Home activities	(In)Dependence participation	Ease/Difficulty participation
Circulation		
Getting in and out of the house	.406^†^	.463^‡^
Going up and down stairs	.421^†^	N.S.
Moving around inside house	N.S.	N.S.
Bathroom		
Getting on and off a toilet	N.S.	.259*
Getting in and out of a bathtub or shower	.314*	.438^‡^
Kitchen		
Using kitchen appliances	.289*	.292*
Getting items in and out of upper cabinets	.417^†^	.320*
Getting items in and out of lower drawers	.272*	N.S.
Bedroom		
Getting on and off a bed	N.S.	N.S.
Getting items in and out of a closet	N.S.	N.S.

*P < .05; ^†^ *P* < .01; ^‡^ *P* < .001.
